# Comprehensive Analysis of YTHDF1 Immune Infiltrates and ceRNA in Human Esophageal Carcinoma

**DOI:** 10.3389/fgene.2022.835265

**Published:** 2022-03-23

**Authors:** Xu-Sheng Liu, Xue-Yan Kui, Yan Gao, Xue-Qin Chen, Jing Zeng, Xiao-Yu Liu, Yu Zhang, Yao-Hua Zhang, Zhi-Jun Pei

**Affiliations:** ^1^ Department of Nuclear Medicine and Institute of Anesthesiology and Pain, Taihe Hospital, Hubei University of Medicine, Shiyan, China; ^2^ Postgraduate Training Basement of Jinzhou Medical University, Taihe Hospital, Hubei University of Medicine, Shiyan, China; ^3^ Hubei University of Medicine, Shiyan, China; ^4^ Department of Infection Control, Taihe Hospital, Hubei University of Medicine, Shiyan, China; ^5^ Hubei Clinical Research Center for Precise Diagnosis and Treatment of Liver Cancer, Taihe Hospital, Hubei University of Medicine, Shiyan, China

**Keywords:** YTH N6-methyladenosine RNA-binding protein 1 (YTHDF1), esophageal carcinoma, immune infiltrates, ferroptosis, ceRNA

## Abstract

**Background:** YTHDF1 is highly expressed in multiple tumors and affects tumor progression. However, there are only a few comprehensive studies on the analysis of YTHDF1 in esophageal cancer.

**Methods:** We analyzed YTHDF1 expression in pan-cancer by comparing both the GEPIA and TCGA cohorts, and further verified the differences in YTHDF1 expression between the ESCA and normal groups by the GEO ESCA cohort and *in vitro* experiments. The correlation of YTHDF1 expression and the clinical characteristics of ESCA patients was analyzed using the TCGA ESCA clinical data. The GO and KEGG enrichment analyses of the YTHDF1 coexpressed genes were completed by bioinformatics analysis, and the GGI and PPI were constructed for the YTHDF1, respectively. The relationship between YTHDF1 expression and the infiltration of ESCA immune cells was analyzed by using the TIMER database and the TCGA ESCA cohort. The relationships between YTHDF1 expression levels and glycolysis and ferroptosis-related genes were analyzed using the TCGA and GEPIA ESCA cohorts. Finally, the ceRNA network that may be involved in YTHDF1 in ESCA was predicted and constructed through a variety of databases.

**Results:** YTHDF1 was overexpressed in various cancers, and *in vitro* experiments confirmed that YTHDF1 expression was higher in ESCA samples than in normal samples. The expression of YTHDF1 has some accuracy in predicting the tumor outcome. Expression of YTHDF1 was significantly associated with multiple clinical features in ESCA patients. GO and KEGG enrichment analyses indicated that YTHDF1 coexpressed genes involved multiple biological functions. There is a potential association between YTHDF1 expression and multiple immune cell infiltration, glycolysis, and ferroptosis-related genes in ESCA. YTHDF1 may be involved in multiple ceRNA regulatory networks in ESCA, including PAXIP1-AS1/hsa-miR-376c-3p/YTHDF1 axis, THUMPD3-AS1/hsa-miR-655-3p/YTHDF1 axis, and SNHG20/hsa-miR-655-3p/YTHDF1 axis, respectively.

**Conclusion:** YTHDF1 can serve as a biomarker of ESCA, related to the immune cell infiltration of ESCA, regulation of glycolysis and ferroptosis, and the ceRNA regulatory network.

## Introduction

The latest research shows that esophageal carcinoma (ESCA) ranks eighth in the global cancer incidence rate, and the death rate ranks sixth (Sung et al., 2021). Although radical resection, radiotherapy, and chemotherapy for ESCA have made significant progress, the 5-year survival rate of ESCA patients is still very low (Kelly, 2019; Yang et al., 2020a; Thrift, 2021). The occurrence, development, and recurrence of ESCA involve a variety of important signal transduction pathways and biological functions in the human body (Yang et al., 2020b; Liu et al., 2021a; Lonie et al., 2021; Thrift, 2021). Therefore, finding biomarkers involving multiple biological functions and exploring the pathogenesis of ESCA can provide a better reference for tumor diagnosis and treatment.

One of the most common internal modifications of mammalian mRNAs is N6-methyladenosine (m6A) RNA modification (Deng et al., 2018; Shi et al., 2019a; Jiang et al., 2021). More and more studies show that m6A modification plays a significant role in regulating the biological function of tumor cells (Wang et al., 2020a). The dynamic imbalance of m6A modification can lead to the occurrence and development of tumors (Deng et al., 2018; Chen et al., 2019). However, YTH N6-methyladenosine RNA-binding protein 1 (YTHDF1) is the core factor of m6A modification (Xu et al., 2021). YTHDF1 can promote the translation of m6A-modified mRNA in cells, thereby regulating translation kinetics (Liu et al., 2020a). It has been reported that YTHDF1 is highly expressed in multiple cancers and is significantly correlated to the development of tumors (Zhao et al., 2018; Bai et al., 2019; Shi et al., 2019b; Liu et al., 2019; Liu et al., 2020b; Wang et al., 2020b). Although some studies found that YTHDF1 was highly expressed in ESCA through bioinformatics analysis (Guo et al., 2021; Zhao et al., 2021), it could not be verified by more data and experiments. The possible biological functions and pathways of YTHDF1 in ESCA have not been widely studied.

Tumor immune cell infiltration, inhibition of glycolysis pathway, regulation of ferroptosis, and regulation of the ceRNA network are an intense focus of research of tumor gene therapies, which are extensively applied in the research and treatment of ESCA (Baba et al., 2020; Liu et al., 2021b; Feng et al., 2021; Lonie et al., 2021; Shi et al., 2021; Shishido et al., 2021). Nevertheless, there area few studies on the thorough study of YTHDF1 in ESCA, especially the relationship between YTHDF1 and ESCA immune cell infiltration, glycolysis, ferroptosis, and the ceRNA network.

**FIGURE 1:**
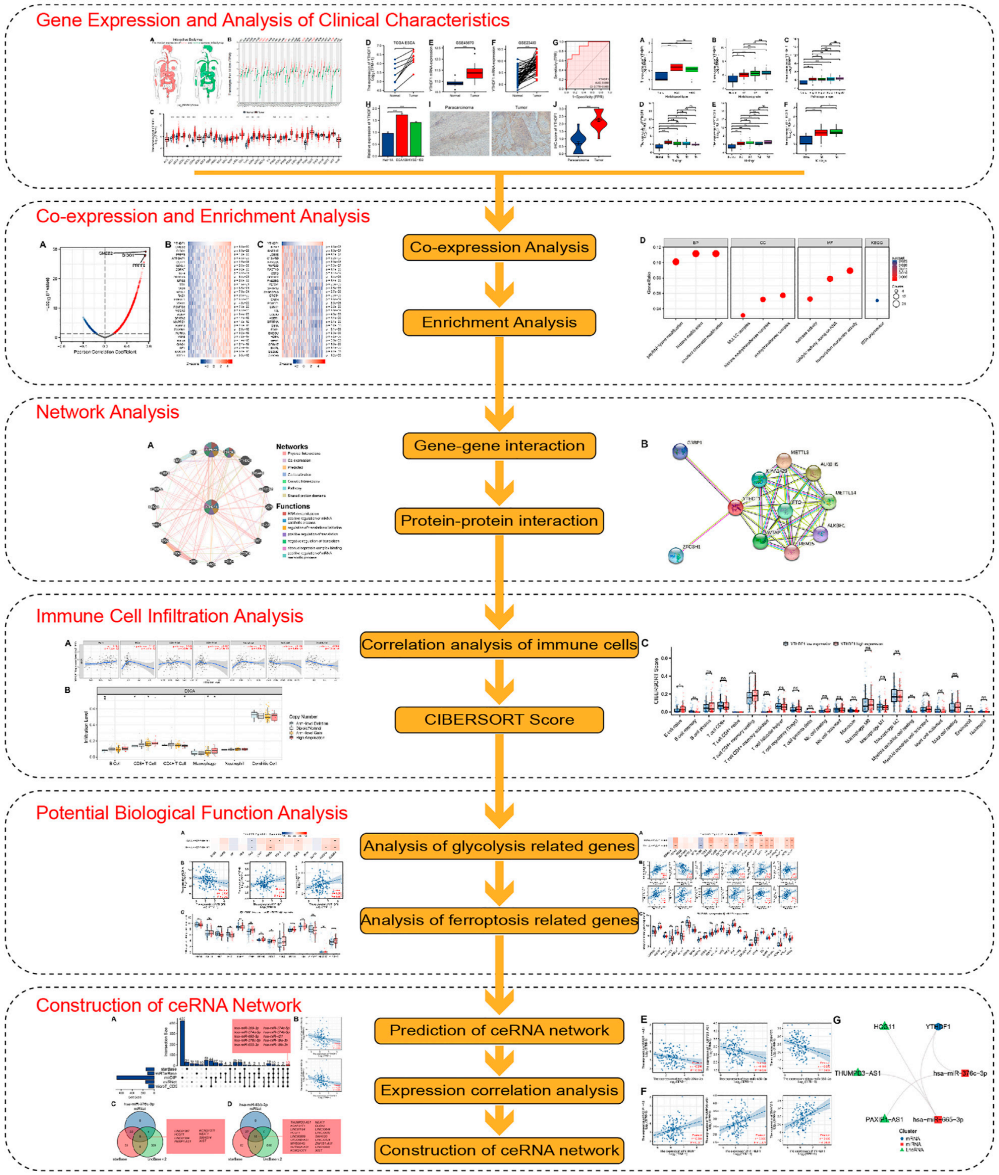
Schematic diagram of the study design.

In the present research, The Cancer Genome Atlas (TCGA) and gene expression synthesis (GEO) dataset cohorts were downloaded and processed. Multiple databases and online websites were analyzed and used. To study the expression difference of YTHDF1 in pan-cancer and verify the expression difference of YTHDF1 mRNA and protein between the ESCA and normal groups by cell experiment and immunohistochemical (IHC) staining experiment, the coexpression gene network of YTHDF1 in ESCA was investigated, and the possible biofunctions and signaling pathways of these genes were studied. Eventually, the relation between YTHDF1 and tumor cell immune infiltration, glycolysis, ferroptosis, and the ceRNA network was surveyed, to provide a basis for the development of new treatment strategies for ESCA. The schematic diagram of the research design is shown in Figure 1.

## Materials and Methods

### Expression of YTH N6-Methyladenosine RNA-Binding Protein 1 in the Esophageal Carcinoma

YTHDF1 expression differences between different tumors and normal samples were analyzed using the Gene Expression Profiling Interactive Analysis (GEPIA, http://gepia.cancer-pku.cn/index.html) (Tang et al., 2017) and TCGA (https://portal.gdc.cancer.gov/) (Tomczak et al., 2015) datasets. GEPIA is a newly developed online website that can analyze RNA expression data for the TCGA and GTEx projects. We downloaded the ESCA cohort from the TCGA and GEO (www.ncbi.nlm.nih.gov/geo; GSE45670 and GSE23400) (Barrett et al., 2012) databases to analyze YTHDF1 expression differences in tumor and normal tissue samples. We also processed clinical data from the ESCA cohort to investigate the relationship of YTHDF1 expression levels and clinicopathological parameters of the ESCA patients. The diagnostic value of YTHDF1 for ESCA was assessed by plotting the ROC curves. Finally, we examined YTHDF1 expression by qRT-PCR and IHC staining assays to analyze its differences in tumor and normal samples. The qRT-PCR and IHC experimental steps were referred to previous studies (Liu et al., 2020c; Liu et al., 2020d). All technical details are provided in the Supplementary Materials.

### YTH N6-Methyladenosine RNA-Binding Protein 1 Gene Coexpression Network and Enrichment Analysis in Esophageal Carcinoma

RNA sequencing data from the TCGA ESCA cohort were analyzed using the STAT package in the R language to study coexpressed genes associated with YTHDF1 expression. The Pearson’s correlation coefficient was used for statistical analysis, and correlations were considered when *p* < 0.05. Volcano maps and heatmaps were drawn using the ggplot2 package in the R language. The top 200 genes positively associated with YTHDF1 expression were selected for subsequent analysis. Gene ontology (GO, http://www.geneontology.org/) term and Kyoto Encyclopedia of Genes and Genomes (KEGG, http://www.genome.jp/kegg) pathway enrichment analyses of the above genes were performed using the clusterProfiler package in the R language (Yu et al., 2012). The results were visualized using the ggplot2 software package in the R language.

### Gene–Gene Interaction and Protein–Protein Interaction Analysis of YTH N6-Methyladenosine RNA-Binding Protein 1

GeneMANIA (www.genemania.org) is an online website that can analyze target genes based on the hypothesis of gene function (Warde-Farley et al., 2010). GeneMANIA can retrieve and create lists of genes functioning similar to the target gene and construct interactive networks. In the present research, we constructed a GGI network of YTHDF1 using GeneMANIA.

STRING (www.string-db.org) is an online website tool that collects and integrates all available data in the public PPI sources (Szklarczyk et al., 2019). In the present research, we constructed the PPI network of YTHDF1 using STRING.

### Correlation Between YTH N6-Methyladenosine RNA-Binding Protein 1 and Tumor Immune Infiltrating Cells

Tumor Immune Estimation Resource (TIMER, https://cistrome.shinyapps.io/timer) is a database for the systematic analysis of various types of tumor immune information (Li et al., 2016; Li et al., 2017). In this study, we used the TIMER database to evaluate the relationship between YTHDF1 expression and immune infiltrating cells in TCGA ESCA cohort. Immune infiltrating cells include B cells, CD8^+^ T cells, neutrophils, CD4^+^ T cells, macrophages, and dendritic cells. At the same time, the somatic copy number alteration (SCNA) module of TIMER tool was performed to connect the genetic copy number variation (CNV) of YTHDF1 with the relative level of tumor infiltrating cells. Taking the median expression of YTHDF1 as the boundary, the TCGA ESCA cohort was divided into two groups: high and low YTHDF1 expression group. The CIBERPORT software package of R language was used to evaluate the relative proportion of 22 immune cells between the high and low YTHDF1 expression groups (Newman et al., 2015).

### Correlation Between YTH N6-Methyladenosine RNA-Binding Protein 1 Expression and Glycolysis-Related Genes in Esophageal Carcinoma

The relation between the expression of YTHDF1 and 14 glycolysis-related genes in TCGA and GEPIA ESCA cohort was analyzed. Relevant genes refer to previous studies (Liu et al., 2021c), including ENO1 (enolase 1), G6PD (glucose-6-phosphate dehydrogenase), HK1 (hexokinase 1), HK2 (hexokinase 2), LDHA (lactate dehydrogenase A), LDHB (lactate dehydrogenase B), PDHB (pyruvate dehydrogenase E1 subunit beta), PDK3 (pyruvate dehydrogenase kinase 3), PDK4 (pyruvate dehydrogenase kinase 4), PGK1 (phosphoglycerate kinase 1), PKM (pyruvate kinase M1/2), SLC2A1 (solute carrier family 2 member 1), SLC2A2 (solute carrier family 2 member 2), and SLC2A3 (solute carrier family 2 member 3). The proportion of 14 glycolysis-related genes between the high and low expression groups of YTHDF1 was analyzed by R software package. Ggplot2 software package was used with the visualization of the data.

### Correlation Between YTH N6-Methyladenosine RNA-Binding Protein 1 Expression and Ferroptosis-Related Genes in Esophageal Carcinoma

The relation between the expression of YTHDF1 and 25 ferroptosis-related genes in TCGA and GEPIA ESCA cohort was analyzed. Relevant genes refer to previous studies (Doll et al., 2019; Liu et al., 2020e), including CDKN1A (cyclin-dependent kinase inhibitor 1A), HSPA5 [heat shock protein family A (Hsp70) member 5], EMC2 (ER membrane protein complex subunit 2), SLC7A11 (solute carrier family 7 member 11), NFE2L2 (nuclear factor, erythroid 2-like 2), MT1G (metallothionein 1G), HSPB1 [heat shock protein family B (Small) member 1], GPX4 (glutathione peroxidase 4), FANCD2 (FA complementation group D2), CISD1 (CDGSH iron sulfur domain 1), FDFT1 (farnesyl-diphosphate farnesyltransferase 1), SLC1A5 (solute carrier family 1 member 5), SAT1 (spermine N1-acetyltransferase 1), TFRC (transferrin receptor), RPL8 (ribosomal protein L8), NCOA4 (nuclear receptor coactivator 4), LPCAT3 (lysophosphatidylcholine acyltransferase 3), GLS2 (glutaminase 2), DPP4 (dipeptidyl peptidase 4), CS (citrate synthase), CARS1 (cysteinyl-TRNA synthetase 1), ATP5MC3 (ATP synthase membrane subunit C locus 3), ALOX15 (arachidonate 15-lipoxygenase), ACSL4 (acyl-CoA synthetase long chain family member 4), and AIFM2 (apoptosis-inducing factor mitochondria-associated 2). The proportion of 25 ferroptosis-related genes between the high and low expression groups of YTHDF1 was analyzed by R software package. Ggplot2 software package was used with the visualization of the data.

### The ceRNA Network of YTH N6-Methyladenosine RNA-Binding Protein 1 was Predicted and Constructed in Esophageal Carcinoma

The miRNAs targeting YTHDF1 were predicted using starBase (https://starbase.sysu.edu.cn/) (Li et al., 2013), miRTarBase (https://mirtarbase.cuhk.edu.cn/) (Huang et al., 2019), mirDIP (https://ophid.utoronto.ca/mirDIP/) (Shirdel et al., 2011; Tokar et al., 2018), miRNet (https://www.mirnet.ca/miRNet/) (Fan et al., 2016; Fan and Xia, 2018; Fan et al., 2018; Chang et al., 2020), and microT_CDS (http://diana.imis.athena-innovation.gr/DianaTools/index.php?r=microT_CDS/index) (Reczko et al., 2012; Paraskevopoulou et al., 2013) databases, respectively. The correlation between YTHDF1 and these miRNAs was analyzed in TCGA ESCA cohort, and the negatively correlated miRNAs were screened as the target miRNAs. The lncRNAs of target miRNAs were predicted using miRNet, starBase, and LncBase v2.0 (http://carolina.imis.athena-innovation.gr/diana_tools/web/index.php?r=lncbasev2%2Findex-predicted) (Paraskevopoulou et al., 2016) databases, the correlation between target miRNAs and these lncRNAs was analyzed in TCGA ESCA cohort, and the negatively correlated lncRNAs were screened as target lncRNAs. In order to further narrow the prediction range, lncRNAs positively correlated with YTHDF1 expression were selected as the final predicted lncRNAs according to the ceRNA theory. The UpSetR (Conway et al., 2017) and igraph software package of R language (Csardi and Nepusz, 2006) were used with the visualization of the data.

## Results

### Pan-Cancer Analysis of YTH N6-Methyladenosine RNA-Binding Protein 1 mRNA Expression in the Gene Expression Profiling Interactive Analysis and The Cancer Genome Atlas

**FIGURE 2:**
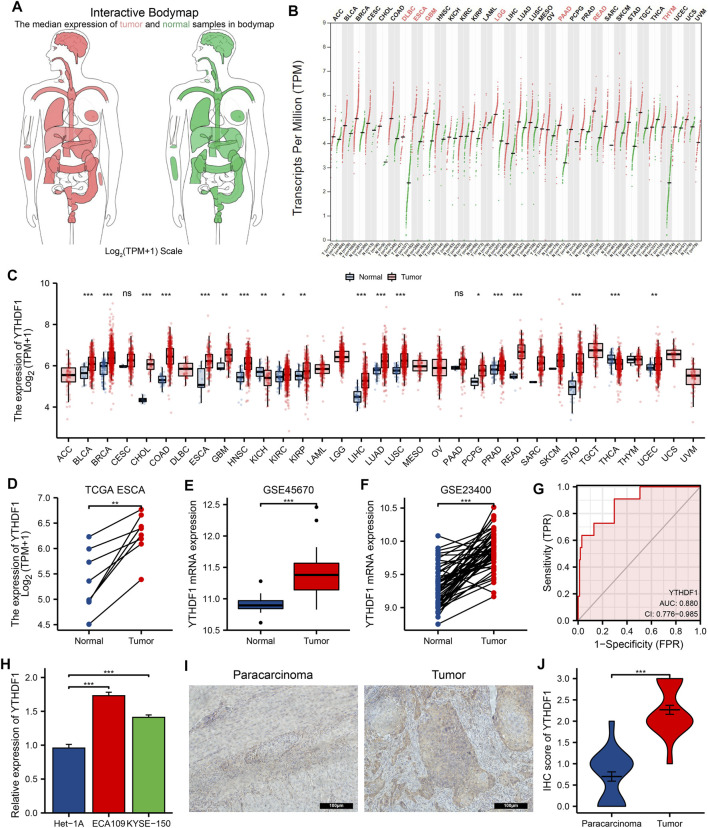
The expression of YTH N6-methyladenosine RNA-binding protein 1 (YTHDF1) in esophageal carcinoma (ESCA) and pan-cancer. **(A)** Body map of YTHDF1 in Gene Expression Profiling Interactive Analysis (GEPIA), red represents YTHDF1 in tumor specimens and green represents YTHDF1 in normal specimens. A darker color indicates a higher gene expression level. **(B)** YTHDF1 expression of tumor and normal tissues in GEPIA. **(C)** The Cancer Genome Atlas (TCGA) cohort summarizes the expression of YTHDF1 in pan-cancer. **(D)** Expression differences of YTHDF1 between paired samples in TCGA ESCA cohort. **(E,F)** The GSE45670 and GSE23400 cohort showed an elevated expression of YTHDF1 in tumor specimens. **(G)** ROC curve analysis of YTHDF1 diagnosis in ESCA. **(H)** Difference in expression of YTHDF1 in ESCA cell lines and human normal esophageal epithelial cell lines. **(I)** The protein expression of YTHDF1 in paracarcinoma and ESCA tissues was estimated by immunohistochemistry. **(J)** The mean YTHDF1 IHC score was significantly higher for the ESCA sample than the matched paracancerous sample. *p < 0.05; **p < 0.01; ***p < 0.001; ns, no significance.

The GEPIA website analysis showed that the YTHDF1 expression was higher in both DLBC (lymphoid neoplasm diffuse large B-cell lymphoma), ESCA, GBM (glioblastoma multiforme), LGG (brain lower grade glioma), PAAD (pancreatic adenocarcinoma), READ (rectum adenocarcinoma), and THYM (thymoma) than in the normal group (Figures 2A, B). Analysis of TCGA dataset showed that YTHDF1 expression was remarkably increased in BLCA (bladder urothelial carcinoma), BRCA (breast invasive carcinoma), CHOL (cholangio carcinoma), COAD (colon adenocarcinoma), ESCA, GBM, HNSC (head and neck squamous cell carcinoma), KIRC (kidney renal clear cell carcinoma), KIRP (kidney renal papillary cell carcinoma), LIHC (liver hepatocellular carcinoma), LUAD (lung adenocarcinoma), LUSC (lung squamous cell carcinoma), PCPG (pheochromocytoma and paraganglioma), PRAD (prostate adenocarcinoma), READ (rectum adenocarcinoma), STAD (stomach adenocarcinoma), and UCEC (uterine corpus endometrial carcinoma), and significantly decreased in KICH (kidney chromophobe) and THCA (thyroid carcinoma) compared with the normal groups, and the analysis results are shown in Figure 2C.

### The Expression Levels of YTH N6-Methyladenosine RNA-Binding Protein 1 in Esophageal Carcinoma Patients

**FIGURE 3:**
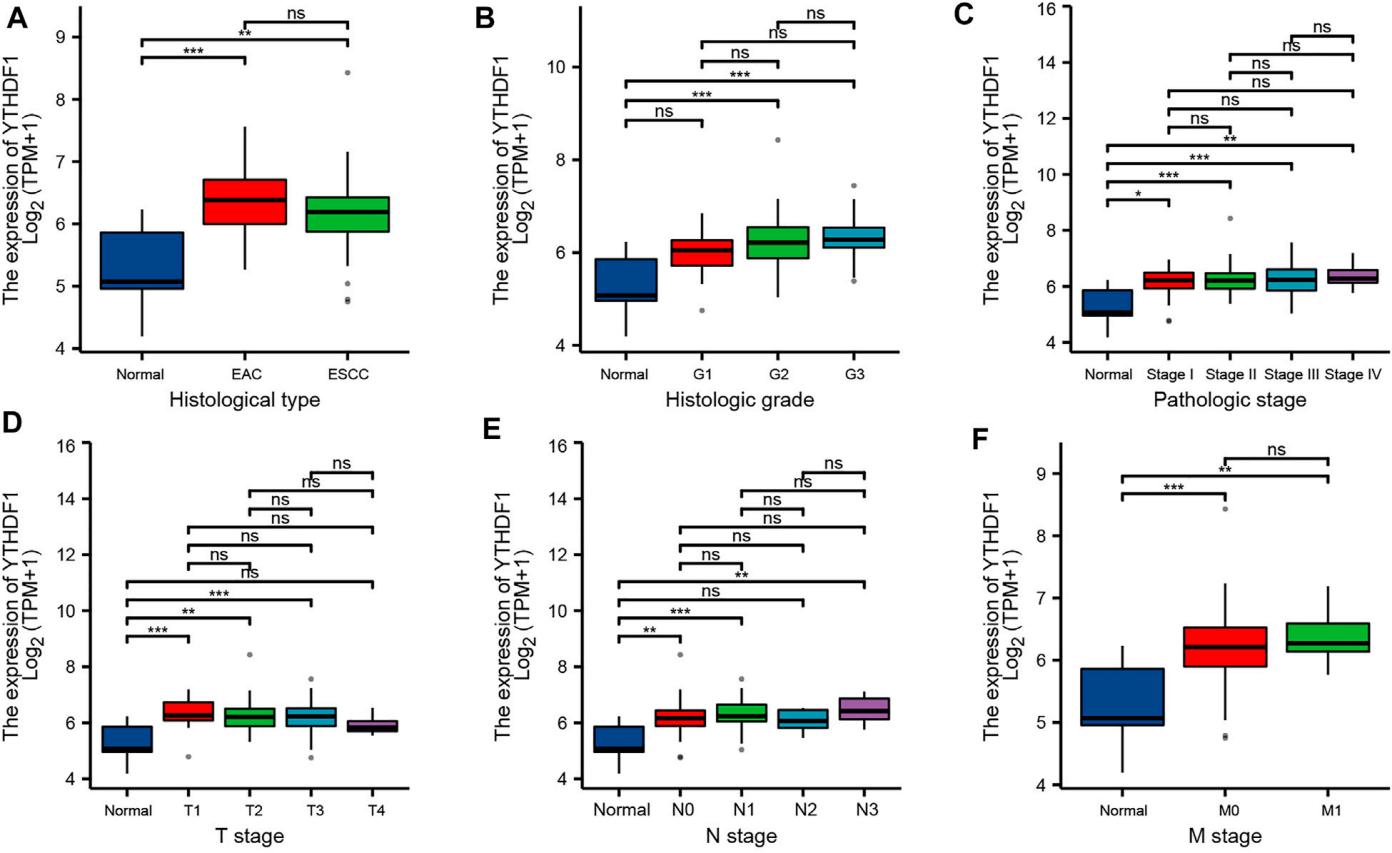
Relationship between YTHDF1 mRNA expression and clinicopathological parameters in esophageal carcinoma (ESCA) patients. Relationship between YTHDF1 mRNA expression level and **(A)** histological type, **(B)** histologic grade, **(C)** pathologic stage, **(D)** T stage, **€** N stage, and **(F)** M stage. *p < 0.05; **p < 0.01; ***p < 0.001; ns, no significance.

By analyzing the ESCA cohort of TCGA and GEO, the results showed that YTHDF1 expression levels were significantly elevated in the ESCA samples compared with the controls (Figures 2D–F). Meanwhile, YTHDF1 expression was further verified by qRT-PCR and IHC staining assays. The qRT-PCR results demonstrated that the expression levels of YTHDF1 mRNA were remarkably increased in ESCA cell lines compared with human normal esophageal epithelial cell line (Figure 2H). IHC staining demonstrated that YTHDF1 was primarily expressed in the cytoplasm (Figure 2I). By calculation, we found that the mean YTHDF1 IHC score was significantly higher in tumor samples than in the paracarcinoma group (Figure 2J). These results suggest that the elevated expression of YTHDF1 may induce the progression of ESCA. Meanwhile, we evaluated the diagnostic potential of YTHDF1 for ESCA by plotting the ROC curves (Figure 2G). The results showed that YTHDF1 has a good prediction accuracy for ESCA, with an area under the ROC curve of 0.880 (95% CI: 0.776–0.985). Finally, we analyzed the clinical data from TCGA ESCA cohort. The results showed that YTHDF1 expression varied in multiple clinical feature groups compared with normal groups, including histological type, histologic grade, pathologic stage, T stage, N stage, and M stage (Figure 3).

### YTH N6-Methyladenosine RNA-Binding Protein 1 Gene Coexpression Network and Enrichment Analysis in Esophageal Carcinoma

**FIGURE 4:**
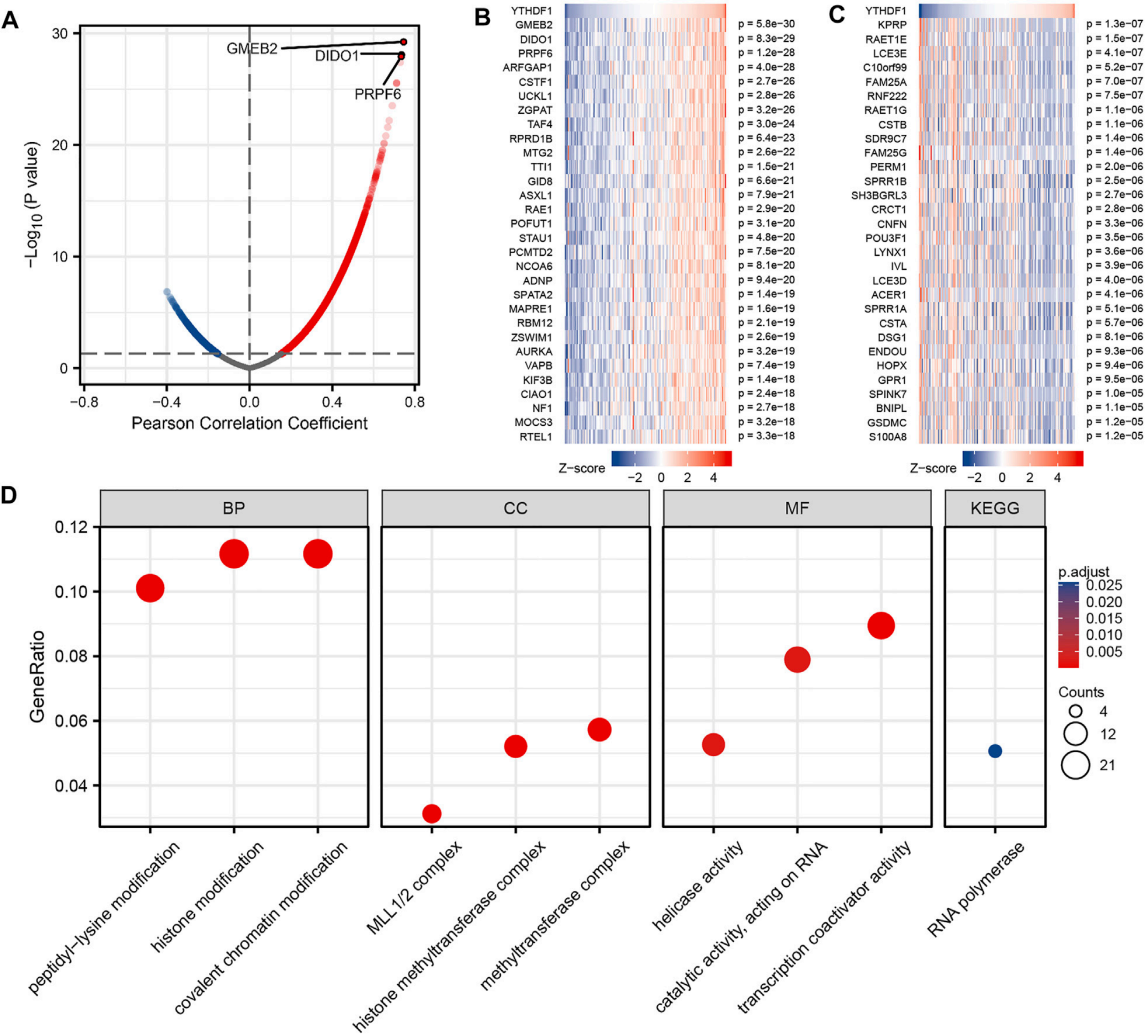
Enrichment analysis of YTHDF1 gene coexpression network in esophageal carcinoma (ESCA). **(A)** Volcano map indicated coexpression genes correlated with the expression level of YTHDF1 in TCGA ESCA cohort. **(B,C)** Heat maps indicated the top 30 coexpression genes positively and negatively associated with expression level of YTHDF1 in the TCGA ESCA cohort. **(D)** Enrichment analysis of Gene Ontology term and Kyoto Encyclopedia of Genes and Genomes (KEGG) pathway for YTHDF1 coexpression genes.

We analyzed the RNA sequencing data from TCGA ESCA dataset using the STAT package in the R language and retained only the genes encoding the protein. The analysis is shown in Figure 4A, a total of 9,412 genes were positively related with YTHDF1 expression, and 628 genes were negatively related with YTHDF1 expression (*p* < 0.05). At |cor| > 0.7 and *p* < 0.05, seven genes were obtained, all of which were positively correlated with YTHDF1 expression. The genes in the top three of the correlation were GMEB2 (glucocorticoid modulatory element-binding protein 2, cor = 0.745, *p* = 5.75E−30), DIDO1 (death inducer–obliterator 1, cor = 0.735, *p* = 8.30E−29), and PRPF6 (preMRNA processing factor 6, cor = 0.733, *p* = 1.19E−28), respectively. We used heat maps (Figures 4B, C) to show the top 30 genes positively and negatively correlated with YTHDF1 expression, respectively. Details of the YTHDF1 coexpressed genes are demonstrated in Supplementary Table S1.

GO term and KEGG pathway analyses of YTHDF1 coexpressed genes were carried using the clusterProfiler package in the R language (Yu et al., 2012). At *p* adj < 0.1 and qvalue < 0.2, YTHDF1 coexpressed genes participated in 169 biological processes, 71 cellular components, 54 molecular functions, and 1 KEGG. Some of the results are presented using bubble plots (Figure 4D). The GO term annotation indicates that these genes are mainly involved in histone modification, histone methyltransferase complex, and transcription coactivator activity. KEGG pathway investigation indicated that these genes are significantly correlated to RNA polymerase. This information descriptions of the results of the YTHDF1 coexpression gene enrichment analysis is summarized in Supplementary Table S3.

### Gene–Gene interaction and Protein–Protein Analysis of YTH N6-Methyladenosine RNA-Binding Protein 1

**FIGURE 5:**
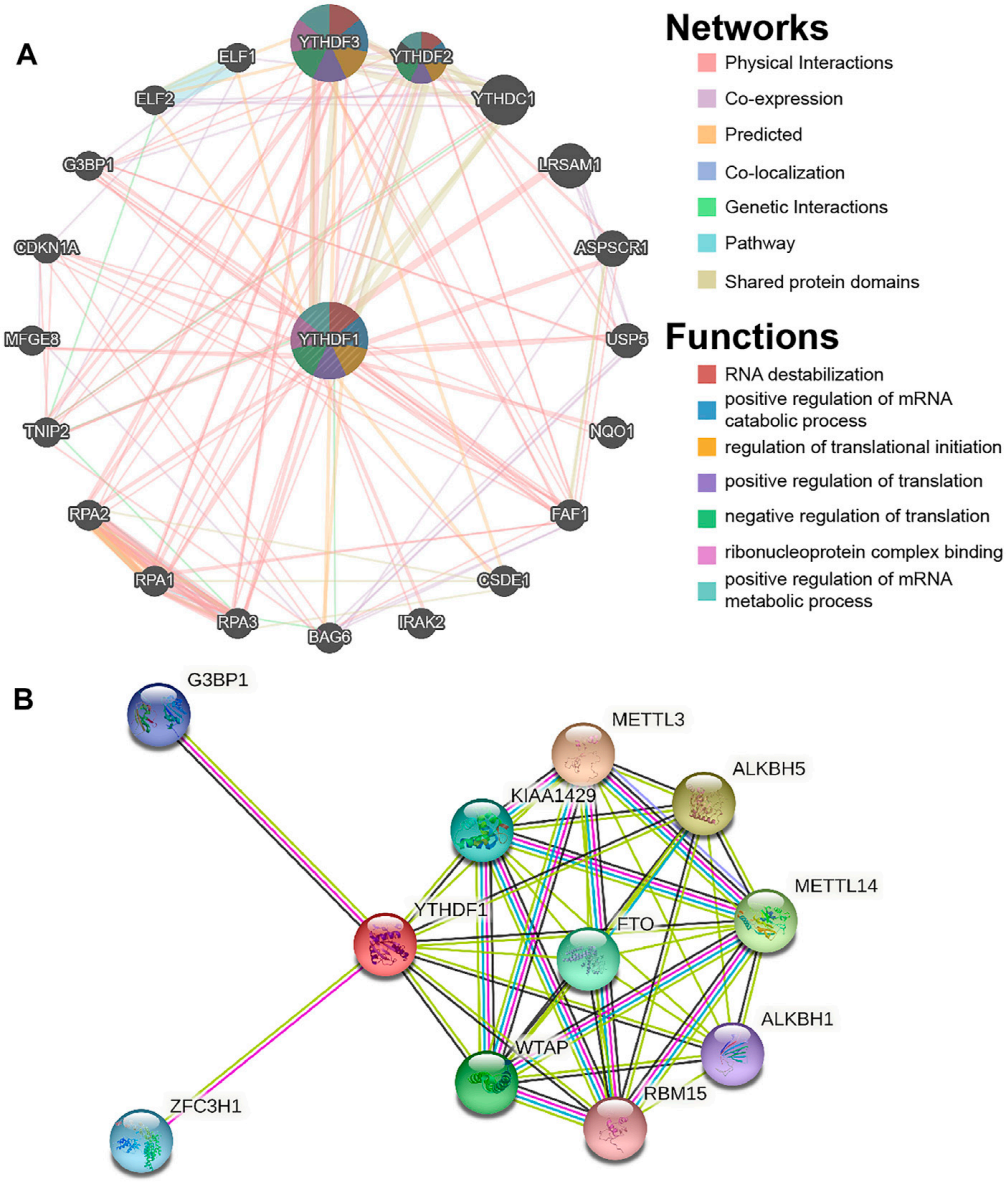
Gene–gene interaction (GGI) and protein–protein interaction (PPI) analysis of the YTHDF1. **(A)** YTHDF1’s GGI network. **(B)** YTHDF1’s PPI network.

The GGI networks of YTHDF1 were constructed using the GeneMANIA database and their functions were analyzed (Figure 5A). The YTHDF1 central nodes are surrounded by 20 nodes that represent genes significantly correlated to YTHDF1 in shared protein domains, genetic interactions, physical interactions, colocalization, coexpression, predicted, and pathways. The top five genes most associated with YTHDF1 are YTHDF3 (YTH N6-methyladenosine RNA-binding protein 3), YTHDF2 (YTH N6-methyladenosine RNA-binding protein 2), YTHDC1 (YTH domain-containing 1), LRSAM1 (leucine-rich repeat and sterile alpha motif-containing 1), and ASPSCR1 (alveolar soft part sarcoma chromosomal region candidate gene 1 protein). Among them, YTHDF1 is associated with YTHDF2 and YTHDF3 in terms of coexpression. YTHDF1 is associated with YTHDF3, ASPSCR1, and LRSAM1 in terms of genetic interactions. YTHDF1 is associated with YTHDF3, YTHDF2, and YTHDC1 in terms of shared protein domains. Further functional analyses suggest that these genes are implicated in many biological functions, including RNA destabilization, positive regulation of mRNA catabolic process, regulation of translational initiation, positive regulation of translation, negative regulation of translation, ribonucleoprotein complex binding, and positive regulation of mRNA metabolic process.

We constructed a PPI network of YTHDF1 using STRING. As demonstrated in Figure 5B, the PPI network map contains YTHDF1 and 10 proteins significantly correlated to them. The three proteins with the highest comprehensive scores were ALKBH5 (AlkB homolog 5), METTL3 (methyltransferase 3), and METTL14 (methyltransferase 14), with scores of 0.95, 0.95, and 0.947, respectively.

### Correlation Between YTH N6-Methyladenosine RNA-Binding Protein 1 and Tumor Immune Infiltrating Cells

**FIGURE 6:**
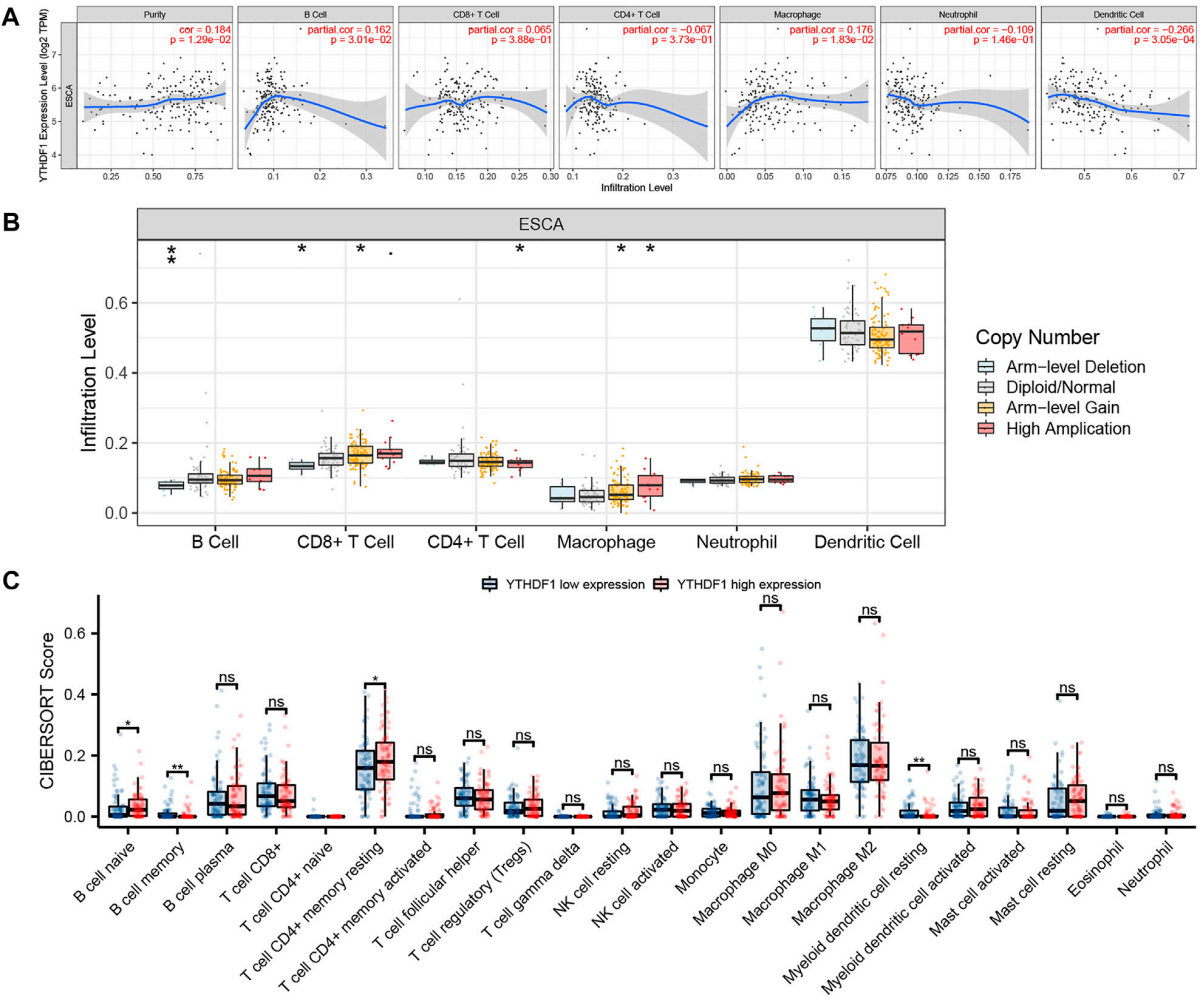
Correlation between YTHDF1 and tumor immune infiltrating cells. **(A)** Correlation between YTHDF1 expression and six types of immune cell infiltration of esophageal carcinoma (ESCA). **(B)** The relation between YTHDF1 copy number variation (CNV) and immune infiltration. **(C)** Changes of 22 immune cell subtypes between the high and low YTHDF1 expression groups in ESCA tumor samples. *p < 0.05; **p < 0.01; ***p < 0.001; ns, no significance.

The relation between the expression level of YTHDF1 in ESCA and six immune infiltrating cells was discussed by using the TIMER database (Figure 6A). The results showed that in the ESCA samples, the expression level of YTHDF1 was positively correlated with the expression levels of B cells (cor = 0.162, *p* = 3.01E−02) and macrophages (cor = 0.176, *p* = 1.83E−02), and negatively associated with the expression level of dendritic cells (cor = −0.266, *p* = 3.05E−04). In addition, YTHDF1 CNV was found to be significantly associated with the infiltration levels of B cells, CD8^+^ T cells, CD4^+^ T cells, and macrophages (Figure 6B, *p* < 0.05). CIBERPORT software package was utilized to investigate the relative proportion of immune cells between the high and low YTHDF1 groups (Figure 6C). The results demonstrated that compared with the low expression group, the relative proportion of B-cell naive (*p* = 0.027) and T-cell CD4^+^ memory resting (*p* = 0.049) increased significantly, while the relative proportion of B-cell memory (*p* = 0.007) and myeloid dendritic cell resting (*p* = 0.006) decreased significantly.

### Correlation Between YTH N6-Methyladenosine RNA-Binding Protein 1 Expression and Glycolysis-Related Genes in Esophageal Carcinoma

**FIGURE 7:**
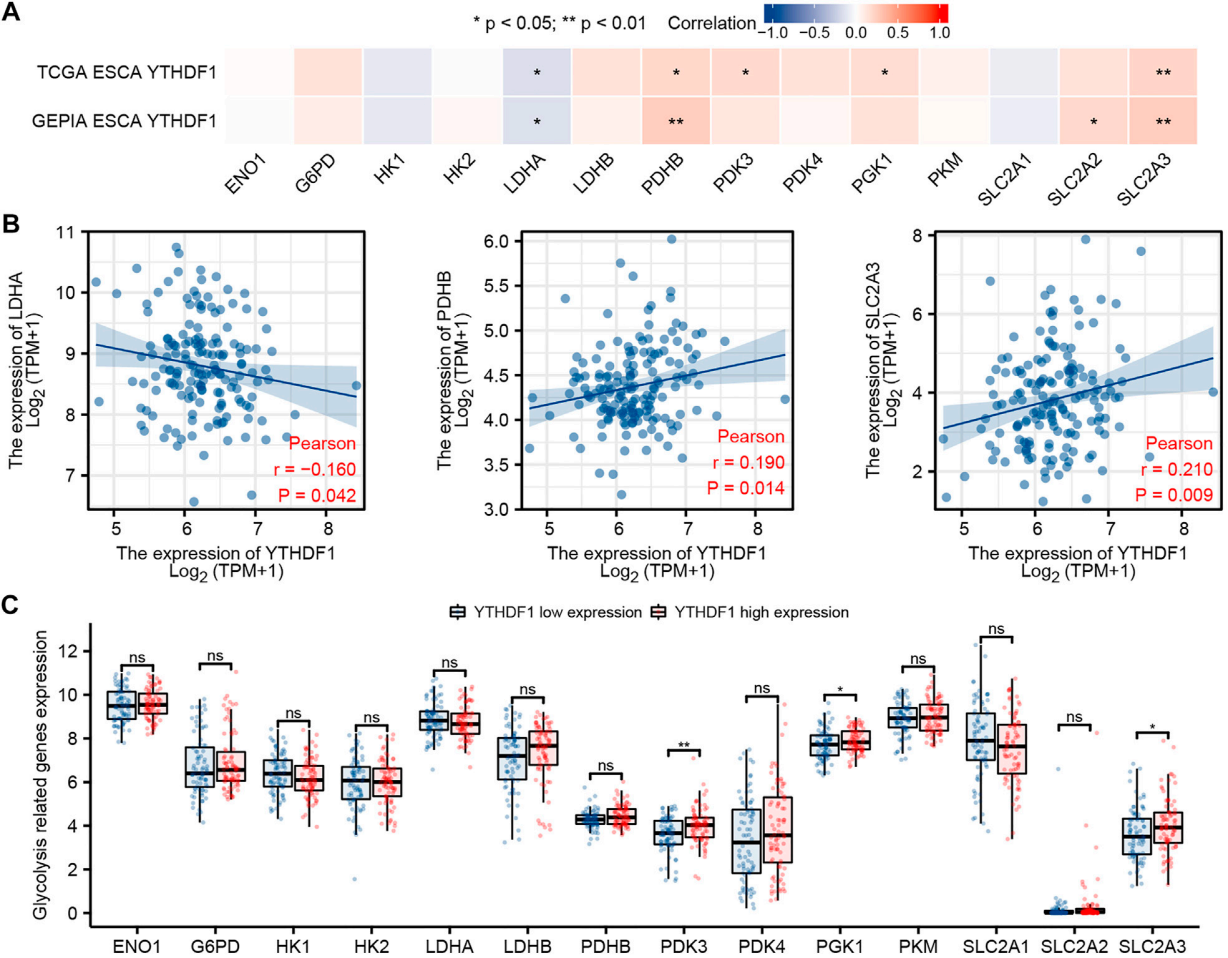
Correlation between YTHDF1 expression and glycolysis-related genes in esophageal carcinoma (ESCA). **(A)** Correlation between expression of YTHDF1 and 14 glycolysis-related genes in TCGA and GEPIA ESCA cohorts. **(B)** Scatter plot demonstrate the correlation between YTHDF1 and three glycolysis-related genes (PDHB, SLC2A3, and LDHA) in TCGA ESCA cohort. **(C)** Differential expression of 14 glycolysis-related genes between the high and low expression groups of YTHDF1 in ESCA. *p < 0.05; **p < 0.01; ***p < 0.001; ns, no significance.

We analyzed the ESCA cohort of TCGA and GEPIA to explore the relation between YTHDF1 and the expression of 14 glycolysis-related genes in ESCA. The results showed that in the ESCA cohort of TCGA and GEPIA, the expression of YTHDF1 was remarkably positively associated with PDHB and SLC2A3, and negatively associated with the expression of LDHA (Figure 7A). In addition, in TCGA ESCA cohort, YTHDF1 expression was remarkably positively associated with PDK3 and PGK1. In the GEPIA ESCA cohort, YTHDF1 expression was significantly positively correlated with SLC2A2. Scatter plots were used to show the association between YTHDF1 and glycolysis-related genes in TCGA ESCA cohort (Figure 7B).

The difference analysis between the high and low groups showed that the expression of PDK3, PGK1, and SLC2A3 increased in the high expression group of YTHDF1 compared with the low expression group (Figure 7C).

### Correlation Between YTH N6-Methyladenosine RNA-Binding Protein 1 Expression and Ferroptosis-Related Genes in Esophageal Carcinoma

**FIGURE 8:**
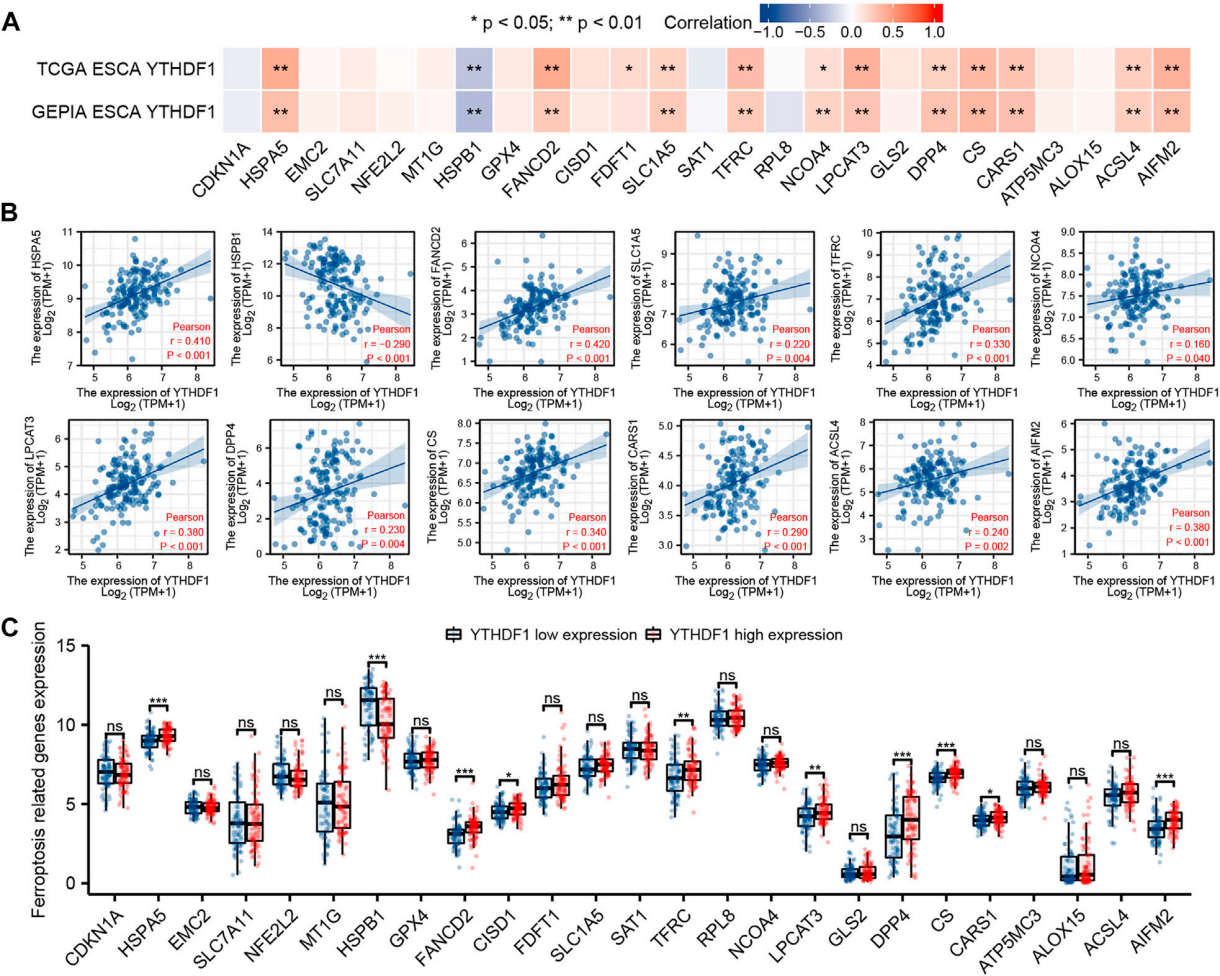
Correlation between YTHDF1 expression and ferroptosis-related genes in esophageal carcinoma (ESCA). **(A)** Correlation between expression of YTHDF1 and 25 ferroptosis-related genes in TCGA and GEPIA ESCA cohorts. **(B)** Scatter plot demonstrate the correlation between YTHDF1 and 12 ferroptosis-related genes (HSPA5, FANCD2, SLC1A5, TFRC, NCOA4, LPCAT3, DPP4, CS, CARS1, ACSL4, AIFM2, and HSPB1) in TCGA ESCA cohort. **(C)** Differential expression of 25 ferroptosis-related genes between the high and low expression groups of YTHDF1 in ESCA. *p < 0.05; **p < 0.01; ***p < 0.001; ns, no significance.

We analyzed the ESCA cohort of TCGA and GEPIA to explore the relation between YTHDF1 and the expression of 25 iron death-related genes in ESCA. The results showed that in the ESCA cohort of TCGA and GEPIA, the expression of YTHDF1 was remarkably positively associated with HSPA5, FANCD2, SLC1A5, TFRC, NCOA4, LPCAT3, DPP4, CS, CARS1, ACSL4, and AIFM2, and negatively associated with the expression of HSPB1 (Figure 8A). In addition, in TCGA ESCA cohort, YTHDF1 expression was remarkably positively associated with FDFT1. Scatter plots were used to show the association between YTHDF1 and ferroptosis-related genes in TCGA ESCA cohort (Figure 8B).

The difference analysis between the high and low expression groups indicated that compared with the low expression group, the expression of HSPA5, FANCD2, CISD1, TFRC, LPCAT3, DPP4, CS, CARS1, and AIFM2 in the YTHDF1 high expression group increased, while the expression of HSPB1 decreased (Figure 8C).

### The ceRNA Network of YTH N6-Methyladenosine RNA-Binding Protein 1 was Predicted and Constructed in Esophageal Carcinoma

**FIGURE 9:**
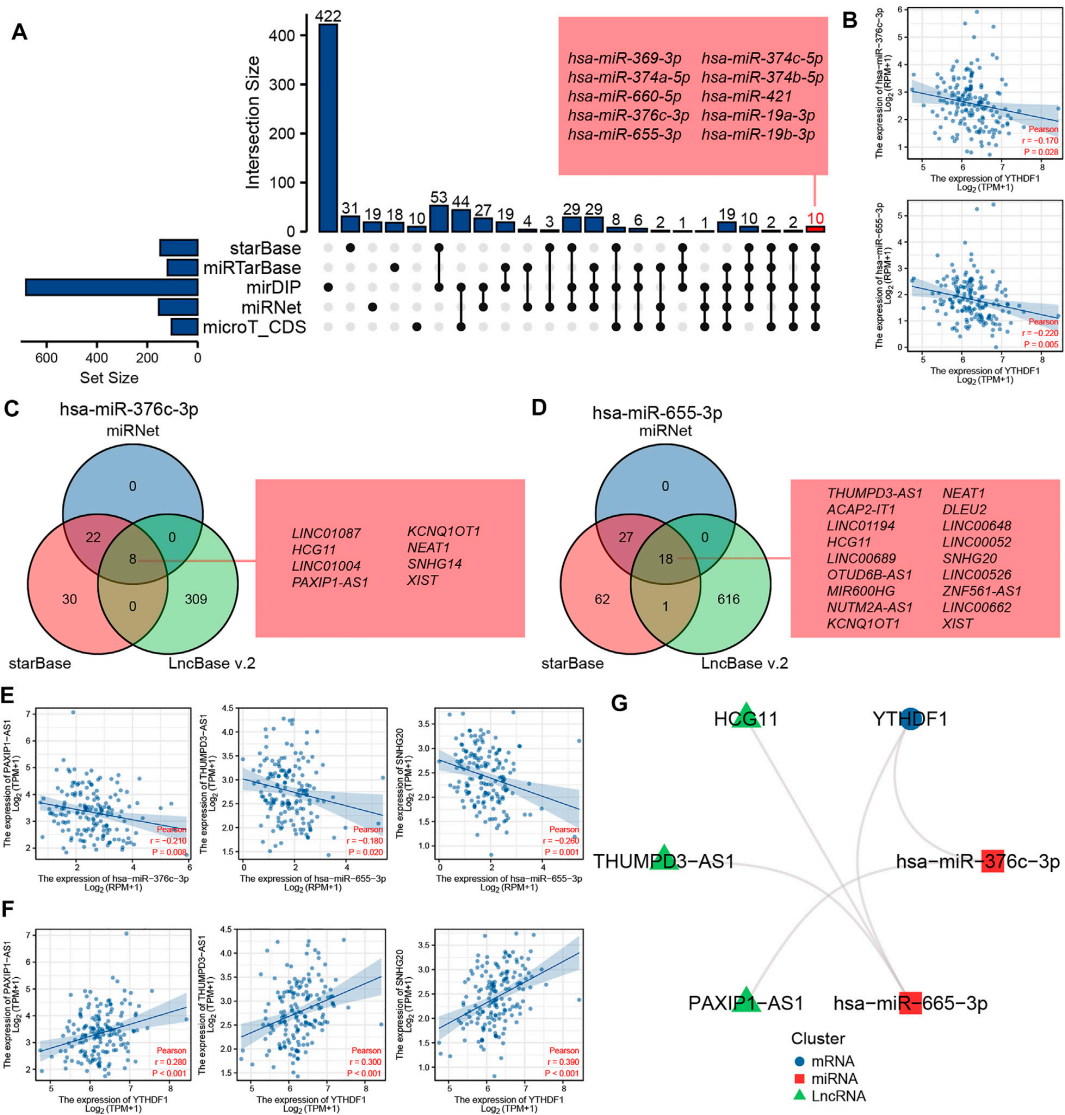
The ceRNA network of YTHDF1 was predicted and constructed in esophageal carcinoma (ESCA). **(A)** The UpSetR diagram displays the miRNA of targeted YTHDF1 predicted by the starBase, miRTarBase, mirDIP, miRNet, and microT_CDS tools. **(B)** The scatter plot shows two miRNAs (hsa-miR-376c-3p and hsa-miR-655-3p) that are significantly negatively correlated with YTHDF1 expression. **(C)** The Venn diagram displays lncRNA of targeted hsa-miR-376c-3p predicted by the miRNet, starBase, and LncBase v2.0 tools. **(D)** The Venn diagram displays lncRNA of targeted hsa-miR-655-3p predicted by the miRNet, starBase and LncBase v2.0 tools. **(E)** The scatter plot shows the lncRNAs that are significantly negatively correlated with the corresponding miRNA expression, respectively. **(F)** The scatter plot shows the lncRNAs that are significantly positively correlated with YTHDF1 expression. **(G)** The network diagram shows the relationship of the final ceRNA network.

We predicted and constructed the ceRNA network of YTHDF1 in ESCA using bioinformatics database. We predicted 149, 120, 682, 155, and 104 miRNAs targeting YTHDF1 using starBase, miRTarBase, mirDIP, miRNet, and microT_CDS tools, respectively. The UpSetR diagram shows the predicted results of the five databases. A total of 10 miRNAs were predicted in all the five databases, and the 10 miRNAs were, hsa-miR-369-3p, hsa-miR-374a-5p, hsa-miR-660-5p, hsa-miR-376c-3p, hsa-miR-655-3p, hsa-miR-374c-5p, hsa-miR-374b-5p, hsa-miR-421, hsa-miR-19a-3p, and hsa-miR-19b-3p, respectively (Figure 9A). In addition, we analyzed the association between these miRNAs and the expression level of YTHDF1 and found that only two miRNAs (hsa-miR-376c-3p and hsa-miR-655-3p) in ESCA were negatively correlated with the expression level of YTHDF1 (Figure 9B).

We predicted lncRNAs that may bind to two miRNAs using miRNet, starBase, and LncBase v2.0 tools, respectively. As shown in Figure 9C, miRNet, starBase, and LncBase v2.0 predicted 30, 60, and 317 lncRNAs targeting hsa-miR-376c-3p, respectively, of which eight lncRNAs were predicted in all the three databases, and the eight lncRNAs were LINC01087, HCG11, LINC01004, PAXIP1-AS1, KCNQ1OT1, NEAT1, SNHG14, and XISTAA, respectively. As shown in Figure 9D, miRNet, starBase, and LncBase v2.0 predicted 45, 108, and 635 lncRNAs targeting hsa-miR-655-3p, respectively, of which 18 lncRNAs were predicted in all the three databases, and the 18 lncRNAs were, THUMPD3-AS1, ACAP2-IT1, LINC01194, HCG11, LINC00689, OTUD6B-AS1, MIR600HG, NUTM2A-AS1, KCNQ1OT1, NEAT1, DLEU2, LINC00648, LINC00052, SNHG20, LINC00526, ZNF561-AS1, LINC00662, and XIST, respectively. Further analysis of the correlation between the expression levels of these lncRNAs and miRNAs showed that there was a negative correlation between the expression levels of PAXIP1-AS1 and miR-376c-3p in ESCA, while there was a negative correlation between the expression levels of THUMPD3-AS1 and SNHG20 and hsa-miR-655-3p (Figure 9E).

In order to further verify the possibility of ceRNA, we analyzed the association between these three lncRNAs and YTHDF1 expression. The results showed that the three lncRNAs were significantly positively correlated with the expression of YTHDF1 (Figure 9F). The complex network diagram shows the network of three ceRNAs, including PAXIP1-AS1/hsa-miR-376c-3p/YTHDF1 axis, THUMPD3-AS1/hsa-miR-655-3p/YTHDF1 axis, and SNHG20/hsa-miR-655-3p/YTHDF1 axis (Figure 9G).

## Discussion

Recent studies have shown that the imbalance of m6A gene modification can lead to a variety of diseases and cancers. YTHDF1 is an m6A-specific RNA-binding protein. Its expression imbalance is significantly correlated with the occurrence and development of many tumors. Shi et al. found that YTHDF1 deficiency can inhibit the proliferation of non-small cell lung cancer cells and the formation of xenograft tumors, and inhibit the progression of *de novo* lung adenocarcinoma (Shi et al., 2019b). Liu et al. found that YTHDF1 was able to enhance EIF3C translation by binding to m6A-modified EIF3C mRNA, thereby promoting tumorigenesis and metastasis in ovarian cancer (Liu et al., 2020b). Bai et al. discovered that knocking out the expression of YTHDF1 could remarkably inhibit the tumorigenicity of CRC cells *in vitro* and the growth of xenograft tumors in mice (Bai et al., 2019). However, the biological functions and pathways that YTHDF1 may participate in ESCA have not been reported.

In this study, we analyzed the data of the GEPIA database and TCGA cohort, and found that YTHDF1 was highly expressed in a variety of tumors. The GEPIA database analysis showed that YTHDF1 was highly expressed in seven cancers. TCGA cohort analysis demonstrated that YTHDF1 was highly expressed in 17 cancers and was low in 2 cancers. The cancer types highly expressed in GEPIA database and TCGA cohort are ESCA, GBM, and READ, which is roughly the same as previous studies (Wang et al., 2020b; Guo et al., 2021). By analyzing TCGA cohort, Guo et al. (Guo et al., 2021) found that YTHDF1 was highly expressed in esophageal squamous cell carcinoma, but failed to use more data for verification. We also analyzed the GEO ESCA cohort and conducted *in vitro* experiments, which further proved that the expression of YTHDF1 in tumor samples was significantly higher than that in normal samples. ROC curve showed that YTHDF1 expression was accurate in predicting ESCA tumor outcome. Finally, it was found that the expression of YTHDF1 was related to histological type, histologic grade, pathological stage, T stage, N stage, and M stage. In conclusion, YTHDF1 can be utilized as a possible diagnostic marker of ESCA.

Presently, the study on the role of YTHDF1 in cancers primarily concentrated on promoting m6A-modified mRNA translation, and there are a few studies on other biological functions that YTHDF1 may involve. This study found that in the coexpression network of YTHDF1, the expression levels of GMEB2, DIDO1, and PRPF6 had the strongest correlation with YTHDF1, but the research of these three genes in ESCA has not been reported. In the future, we plan to further verify the potential relationship between YTHDF1 and these three genes through more experiments. GO and KEGG analysis indicated that the coexpression of YTHDF1 was primarily related to histone modification, histone methyltransferase complex, and transcription coactivator activity. KEGG pathway analysis indicated that the coexpression of YTHDF1 was primarily related to RNA polymerase. However, these biological functions and pathways are closely related to tumor development. Kar et al. found active histone modifications to be a determinant of the induced overexpression of OCT4 during breast carcinogenesis (Kar and Patra, 2018). Wei et al. found that the expression of G9a histone methyltransferase was upregulated in human hepatocellular carcinoma (HCC), which led to the epigenetic silencing of HCC suppressor gene RARRES3, and finally enhanced the proliferation and migration of HCC cells (Wei et al., 2017). Urban et al. found that the expression of transcription coactivator BCl3 increased in human CRC and promoted the proliferation of mouse xenografts *in vivo* and the survival of tumor cells *in vitro* (Urban et al., 2016). Bywater et al. found that inhibition of RNA polymerase can induce the cancer-specific activation of p53 and achieve the purpose of tumor inhibition (Bywater et al., 2012). This indicates that the coexpression network of YTHDF1 plays an important role in tumor proliferation and development.

GGI network analysis discovered that YTHDF1 was closely associated with YTHDF3 and YTHDF2, which may be because their biological functions are similar and are mainly used to identify the information of m6A methylation in the cytoplasm (Liu et al., 2020a; Xu et al., 2021). Cui et al. found that the increase of FTO in esophageal squamous cell carcinoma (ESCC) inhibited the decay of LINC00022 through m6A reader YTHDF2, thus, promoting cell cycle progression and proliferation (Cui et al., 2021). However, there is no report on the mechanism of YTHDF1 and YTHDF3 promoting ESCA. In the future, we will carry out more mechanism research on this problem. The PPI network found that YTHDF1 had the highest comprehensive scores with ALKBH5, METTL3, and METTL14. However, there are different conclusions about the study of ALKBH5 in ESCA. Chen et al. discovered that ALKBH5 inhibited the malignant proliferation of ESCA by regulating microRNA biogenesis and RAI1 expression (Chen et al., 2021a). Nagaki et al. found that ALKBH5 promoted the proliferation of ESCC and was associated with poor prognosis (Nagaki et al., 2020). This interesting discovery deserves further discussion. In our previous studies, METTL3 was found to be highly expressed in ESCA and may be potentially associated with glycolysis of tumor cells (Liu et al., 2020d). Liu et al. found that the METTL14/mir-99a-5p/tribble 2 positive feedback pathway can promote the persistence and radiation resistance of ESCC stem cells (Liu et al., 2021d). The study of these related genes further confirmed the possibility of YTHDF1 participating in the progression of ESCA.

Tumor immune cell infiltration is an important component of the tumor immune microenvironment (TIME) (Baba et al., 2020). The results showed that the expression of YTHDF1 was positively correlated with the infiltration of B cells and macrophages, but negatively correlated with the infiltration of dendritic cells. YTHDF1 CNV was significantly correlated with the infiltration levels of B cells, CD8^+^ T cells, CD4^+^ T cells, and macrophages B cells. However, compared with the low YTHDF1 expression group, the B-cell naive and T-cell CD4^+^ memory resting infiltration increased, while the B-cell memory and myeloid dendritic cell resting infiltration decreased in the high YTHDF1 expression group. Chen et al. found that compared with the control group, the expression of CD8 and CD86 in tumor tissue and peripheral blood of ESCA patients decreased significantly, suggesting that impaired immune function and reduced number of dendritic cells are the potential causes of the occurrence and development of ESCA (Chen et al., 2004). So, we believe that YTHDF1 expression may affect TIME regulation, especially affecting the immune response within the body by influencing dendritic cells infiltration.

The enhancement of glycolysis pathway can provide sufficient energy for the formation of tumor cells, and then enhance the proliferation and development of tumor cells (Liu et al., 2021b). Previous studies have shown that YTHDF1 expression can promote the glycolytic ability of LUAD (Yang et al., 2021) and cervical cancer (Wang et al., 2020c) cells, so as to enhance the proliferation of tumor cells. However, the effect of YTHDF1 on glycolysis of ESCA has not been reported in ESCA. In this study, we discovered that YTHDF1 was significantly positively correlated with the expression of glycolysis-related genes PDHB and SLC2A3. However, the expression level of SLC2A3 in the YTHDF1 high expression group was significantly higher than that in the YTHDF1 low expression group. Pan et al. found that LINC00667 plays a critical role in metastatic ESCA by mediating sponge regulatory axis miR-200b-3p/SLC2A3 (Pan and Zang, 2021). Therefore, we believe that high expression of YTHDF1 may promote the expression of SLC2A3, promote the glycolysis of ESCA, and finally promote the progress of cancer.

The discovery of a new regulatory cell death process, ferroptosis, has made progress in cancer treatment. However, ferroptosis plays a dual role of promoting and inhibiting cancer in cancer progression (Chen et al., 2021b). It has been found that the abnormal expression of ferroptosis-related genes can regulate the ferroptosis process in tumor cells and ultimately affect the progress of ESCA. In this study, we found that YTHDF1 was potentially associated with multiple ferroptosis-related genes. There was a positive relation with the expression level of YTHDF1, and the genes overexpressed in the YTHDF1 high expression group were HSPA5, FANCD2, TFRC, LPCAT3, DPP4, CS, CARS1, and AIFM2. However, there was a negative correlation with the expression level of YTHDF1, and the gene low expression in the YTHDF1 high expression group was HSPB1. However, there are a few studies on the effect of ferroptosis on ESCA. This interesting result of YTHDF1 expression associated with multiple ferroptosis-related genes is a potential help for subsequent studies that YTHDF1 affects ESCA progression by regulating ferroptosis.

A newly discovered mechanism of mutual regulation between RNAs, the ceRNA regulatory network, is widely present and plays important roles in different tumors (23, 24). In this study, we predicted and constructed a ceRNA regulatory network involving YTHDF1 in ESCA, which were PAXIP1-AS1/hsa-miR-376c-3p/YTHDF1 axis, THUMPD3-AS1/hsa-miR-655-3p/YTHDF1 axis, and SNHG20/hsa-miR-655-3p/YTHDF1 axis, respectively. It has been found that PAXIP1-AS1 (Ma and Zheng, 2021), THUMPD3-AS1 (Hu et al., 2019), SNHG20 (Wu et al., 2019), hsa-miR-376c-3p (Lin et al., 2019), and hsa-miR-655-3p (Xin et al., 2020) all participate in the ceRNA regulatory network and play a specific role in different tumors. However, there are few studies on these RNAs in ESCA, especially the relationship with YTHDF1 has not been reported in ESCA. Our findings provide potential support for the involvement of YTHDF1 in ceRNA regulatory networks affecting ESCA progression. However, more basic experiments are needed to further prove our evaluation in the future.

In conclusion, this is the first comprehensive investigation of the relation between YTHDF1 expression and ESCA tumor cell immune infiltration, glycolysis, ferroptosis, and the ceRNA regulatory network. YTHDF1 expression is correlated to multiple immune infiltrating cells, which may impact the tumor immunity of ESCA by affecting the infiltration of dendritic cell. YTHDF1 has a strong correlation with glycolysis-related gene SLC2A3, which may impact the glycolysis pathway of ESCA by affecting the expression of SLC2A3. However, YTHDF1 is potentially associated with multiple ferroptosis-related genes, which may affect the regulation of ferroptosis in tumor cells by affecting the expression of these genes. Finally, we also found that YTHDF1 may affect the progress of ESCA by participating in multiple ceRNA regulatory networks. YTHDF1 can be utilized as a possible biomarker for ESCA diagnosis and treatment. Of course, we will further verify the results of our analysis through more experiments in the future.

## Data Availability

The original contributions presented in the study are included in the article/Supplementary Material. Further inquiries can be directed to the corresponding author.
